# First epidemiological survey of *Angiostrongylus vasorum* in domestic dogs from Spain

**DOI:** 10.1186/s13071-020-04180-5

**Published:** 2020-06-12

**Authors:** Elena Carretón, Rodrigo Morchón, Yaiza Falcón-Cordón, Jorge Matos, Noelia Costa-Rodríguez, José A. Montoya-Alonso

**Affiliations:** 1 Internal Medicine, Faculty of Veterinary Medicine, Research Institute of Biomedical and Health Sciences (IUIBS), University of Las Palmas de Gran Canaria, Las Palmas de Gran Canaria, Spain; 2 Animal and Human Dirofilariosis Group, Laboratory of Parasitology, Faculty of Pharmacy, University of Salamanca, Campus Miguel Unamuno s/n, 37007 Salamanca, Spain

**Keywords:** Domestic dogs, Canine angiostrongylosis, *Angiostrongylus vasorum*, Prevalence, Epidemiology, Spain

## Abstract

**Background:**

*Angiostrongylus vasorum* is the causative agent of canine angiostrongylosis, a disease that mainly affects domestic dogs and other wild carnivores. In Europe, the number of infected individuals is increasing, being located in central and southern countries. In Spain, several studies have reported high prevalence of *A. vasorum* in wild animals. However, there are no studies addressing the current situation of the disease or its distribution in domestic dogs, and reports from veterinary personnel are very limited. Considering these facts, the objective of the present study was to evaluate the prevalence of *A. vasorum* in different areas of Spain.

**Methods:**

Between November 2018 and October 2019, blood was sampled from a total of 2024 domestic dogs from six zones of Spain with a climate that favours the establishment of the disease, where all dogs included in the study lived outdoors or had regular access to areas with vegetation and none had travelled outside the study area of interest in the past year. Details about their sex and age were collected. All dogs were tested for the presence of *A. vasorum* circulating antigens using Angio Detect^TM^.

**Results:**

The overall prevalence of canine angiostrongylosis in the studied areas of Spain was 1.73%. No differences in overall prevalence were found between males and females, neither between age groups. Regarding eco-epidemiological areas, the highest prevalence was recorded in the zones located in the north and northwest of Spain (1.86–2.74%), which correspond to the wetter climates and most abundant vegetation, and the lowest prevalence was detected in the zones located in the center and west of Spain (0.93–0.99%).

**Conclusions:**

Our data suggest that angiostrongylosis is present in Spain in domestic dogs where previously infected wild animals existed or where climatic conditions are favourable for the establishment of the disease.
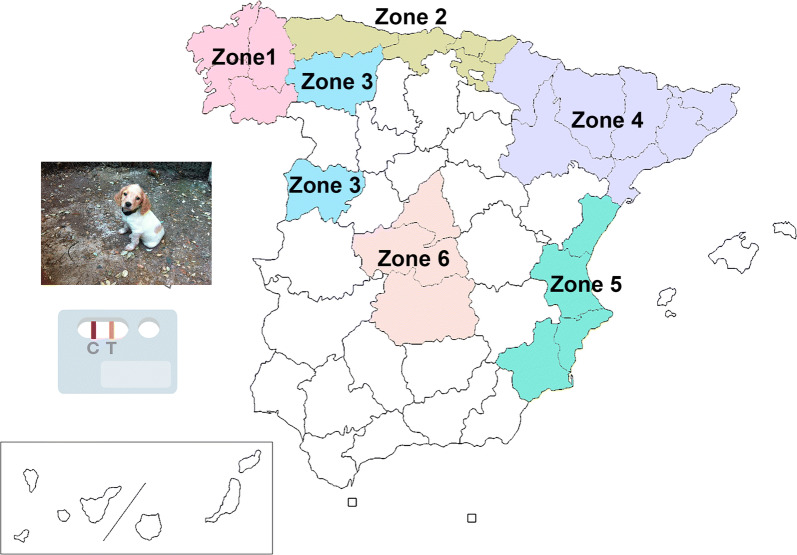

## Background

*Angiostrongylus vasorum* is the causative agent of canine angiostrongylosis. It is a metastrongyloid nematode that mainly affects domestic dogs (*Canis lupus*) and wild carnivores such as foxes (*Vulpes vulpes*), wolves (*Canis lupus*), golden jackals (*Canis aureus*) and others in Europe [1]. Adult worms reside in the right side on the heart and the pulmonary arteries of the definitive host. Female worms shed first-stage larvae (L1) that are released in faeces. These are ingested by gastropod molluscs [2] and develop to L3; amphibians and birds can also act as paratenic hosts. When the definitive hosts feed on intermediate or paratenic hosts, L3 larvae penetrate through the intestinal wall, and migrate to the mesenteric lymph nodes where they mature to L5; once that stage is reached, larvae migrate to the right ventricle and pulmonary arteries through the mesenteric lymphatic vessels, portal vein, hepatic veins and caudal vena cava. The prepatent period ranges from 1 to 2 months [3]. Currently, *A. vasorum* is not considered zoonotic [4]. The parasite can cause a wide range of clinical signs in dogs, and three main clinical manifestations have been described: cardiorespiratory, hemorrhagic diathesis and neurological disease, while some dogs may remain asymptomatic. The severity of some clinical signs can lead to the death of the host. The unspecific nature of the symptoms makes it difficult to diagnose this disease, and this is one of the reasons why canine angiostrongylosis is underdiagnosed [4, 5].

In Europe angiostrongylosis is considered an emerging disease, reported in southern France, Austria, Denmark, France, Greece, Germany, Hungary, Ireland, Italy, Portugal, Romania, Spain, Sweden, Switzerland, Turkey, and the UK, mainly in foxes and reaching a prevalence of 80% in some regions [1, 6–10]. In Spain, *A. vasorum* has been mainly recorded in foxes, with prevalence ranging between 1.8–43.2%, in the north (Asturias and Basque country), northwest (Galicia), west (Castile and León), northeast (Catalonia and Aragon) and east (Murcia) [11–16]. Other species with records of *A. vasorum* are the Iberian wolf in the north and north-west (Asturias, Galicia, and Castile and León) with prevalence ranging between 2.1–21.6% [12, 17], badgers in all the Mediterranean area (6.4%) [18], and one dog [19]. *Angiostrongylus daskalovi* has been reported in the Basque Country in badgers (prevalence of 24%) [15].

There are different techniques to carry out a specific diagnosis. Baermann’s method for detecting L1 in stool samples is considered the gold standard; in addition, the FLOTAC technique has great sensitivity [20]. However, these techniques have several drawbacks, for example, larvae of *A. vasorum* can be mistaken for other larvae of lung worms (*Filaroides* spp.) or larvae of free-living nematodes, unless morphological identification is performed by an experienced veterinarian. There are other specific methods, such as PCR detection and serological methods, which are used for epidemiological screening. A commercial blood test for the detection of *A. vasorum* antigens in domestic dogs with high sensitivity and specificity is currently available [1].

Given the presence and, in some areas, high prevalence of *A. vasorum* in wild carnivores, as well as the absence of studies in domestic dogs, the objective of this study was to determine the prevalence of canine angiostrongylosis in dogs in various areas of Spain.

## Methods

### Climatic characteristics of the study areas

The temperate Mediterranean climate predominates in Spain. In the northern regions, rainfall and vegetation are abundant. In the eastern and southern regions, rainfall is scarce, so the vegetation is much scarce than in the north. In the west, only winters are rainy.

**Fig. 1:**
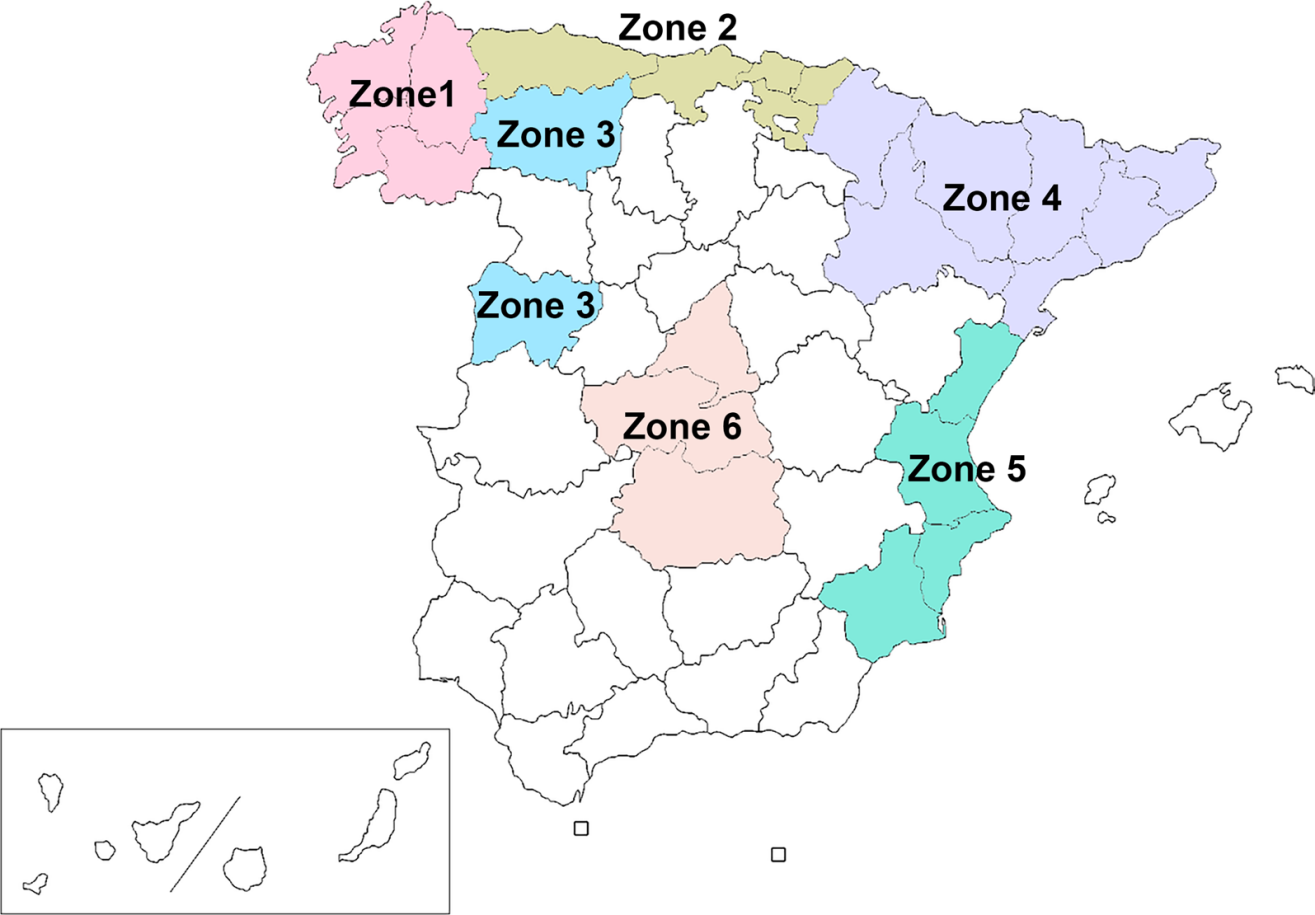
Map of Spain showing the geographical location of the six sampled zones

The survey was carried out in six different eco-epidemiological areas of Spain (Fig. 1), and based on the Köppen Climate Classification [21]: (i) Zone 1, comprising the autonomous region of Galicia in the northwest, has a temperate with dry or temperate summer climate (Csb), with rainy winters and a minimum of rainfall during the summer, and temperature of the warmest month not exceeding 22 °C; (ii) Zone 2, including the autonomous regions of Asturias, Cantabria and Basque Country, all located in the north. The climate is mainly temperate with a dry season and temperate summer climate (Cfb), with abundant rainfall throughout the year and the temperature of the warmest month not exceeding 22 °C; (iii) Zone 3, including the provinces of Salamanca and León, in the west; the Csb climate predominates, although areas of temperate with dry or hot summer climate (Csa) are also present, the latter is characterized by having hot summers with the temperature of the warmest month > 22 °C; (iv) Zone 4, in the northeast comprising the autonomous regions of Catalonia and Navarra, as well as the provinces of Zaragoza and Huesca; this zone has a varied climate, including cold climates (type D) in the Pyrenean area, type C climates: Cfb, Csa, and temperate with a dry season and hot summer (Cfa). Also, cold steppe climate (Bsk) is present, which corresponds to a dry climate characterized by evaporation exceeding precipitation on average but is less than potential evaporation, and the average annual temperature < 18 °C; (v) Zone 5, in the east, including the autonomous regions of Valencian Community and Murcia; where Csa and Bsk are the predominant climates; (vi) Zone 6, located in the centre and comprising the autonomous region of Madrid and the provinces of Toledo and Ciudad Real with Csa being the predominant climate, but also there are significant extensions of Bsk climate.

### Sample collection and analysis

Between November 2018 and October 2019, a total of 2024 domestic dogs from the six zones of Spain were included in the study. Of these, 323 came from Zone 1, 438 from Zone 2, 216 from Zone 3, 539 from Zone 4, 407 from Zone 5, and 101 from Zone 6. Age and sex were recorded for each dog.

The criteria for inclusion in the study were: being over 3 months of age; never having received prophylactic treatment against *A. vasorum*; no previous history of angiostrongylosis; and owner consensus to participate in the survey. All of the studied dogs lived outdoors or had regular access to areas with vegetation and none had travelled outside the study area of interest in the past year.

Blood samples collected from the cephalic or jugular vein were placed in 3 ml serum tubes and centrifuged at 1800× *g* for 10 min. Serum samples were kept at -20 °C until tests were performed. All samples were tested at the University of Salamanca, Spain, for the presence of *A. vasorum* circulating antigens using Angio Detect™ (IDEXX Laboratories Inc., Westbrook, Maine, USA) following manufacturer’s instructions.

### Statistical analysis

Data were analysed using SPSS Base 20.0 software for Windows (SPSS Inc./IBM, Chicago, Illinois, USA). Descriptive analysis of the considered variables was carried out considering the proportions of the qualitative variables. Chi-square test and Fisher’s exact test were used to compare proportions. In all cases, the significance level was established at *P* < 0.05.

## Results

The overall prevalence of canine angiostrongylosis in the studied areas of Spain was 1.73%, with no statistically significant differences between female and male dogs (1.70% and 1.66%, respectively). When the data were broken down by zone (Table 1), the prevalence of males and females was similar and with no statistically significant differences between them, except in zones 3 and 6, where all males or females, respectively, were uninfected; however, the differences related to sex for these two zones were also not statistically significant (Table 1).

**Table 1:** Canine prevalence of *Angiostrongylus vasorum* by age and sex from six zones of Spain

Zone		1	2	3	4	5	6	Total
		*n*/*N* (%)	*n*/*N* (%)	*n*/*N* (%)	*n*/*N* (%)	*n*/*N* (%)	*n*/*N* (%)	*n*/*N* (%)
Age (years)	< 1	1/21 (4.76)	2/19 (10.53)	0/9 (0)	1/47 (2.13)	0/32 (0)	0/1 (0)	4/129 (3.10)
	1–4	3/71 (4.23)	6/98 (6.12)	1/44 (2.27)	3/191 (1.57)	2/102 (1.96)	0/33 (0)	15/539 (2.78)
	5–10	1/123 (0.81)	2/191 (1.05)	1/68 (1.47)	2/164 (1.22)	3/172 (1.74)	1/42 (2.38)	10/760 (1.32)
	> 10	1/108 (0.93)	2/130 (1.54)	0/95 (0)	1/137 (0.73)	2/101 (1.98)	0/25 (0)	6/596 (1.01)
Sex	Male	2/126 (1.58)	6/220 (2.73)	0/80 (0)	3/227 (1.32)	4/195 (2.05)	1/65 (1.54)	16/963 (1.66)
	Female	4/197 (2.03)	6/218 (2.75)	2/136 (1.47)	4/312 (1.28)	3/212 (1.42)	0/42 (0)	19/1067 (1.70)
Total		6/323 (1.86)	12/438 (2.74)	2/216 (0.93)	7/539 (1.30)	7/407 (1.72)	1/101 (0.99)	35/2024 (1.73)

The mean age of the infected dogs was 5.66 years-old, with infected dogs ranging from 5 months-old to 10 years-old. The highest overall prevalence was recorded in younger dogs (< 1 year-old) (3.10%) and there was a gradual decrease in the subsequent age groups until reaching the lowest overall prevalence in older dogs (> 10 years-old) (1.01%). No significant differences in overall prevalence were found between age groups. Evaluation of the age groups by zones revealed that the prevalences of angiostrongylosis in dogs < 4 years-old are higher in Zones 1 and 2, while in Zone 6 all < 4 years-old dogs were uninfected (Table 1). The only significant differences between age groups were found in Zone 2 (*χ*^2^ = 11.15, *df* = 3, *P* = 0.011), between < 1-year-old and 5–10 years-old dogs (OR = 0.09, 95% CI: 0.01–0.61, *P* = 0.042), and between 1–4 years-old and 5–10 years-old dogs (OR = 0.16, 95% CI: 0.03–0.67, *P* = 0.021) (Table 1).

By eco-epidemiological areas, the highest overall prevalence was recorded in Zone 2 (2.74%) and Zone 1 (1.86%), corresponding to the regions that have the rainiest climates and coolest summers compared to the rest of the climates. The lowest prevalences corresponded to Zones 6 and 3 (0.99% and 0.93%, respectively), corresponding to climates characterized by hot and dry summers (Table 1). No significant differences were found in the overall prevalence between zones.

## Discussion

In Europe, *A. vasorum* has been reported both in wild animals (foxes, wolves, badgers) and in domestic dogs, being considered an emerging parasite [3]. Until two decades ago, angiostrongylosis was a disease only recorded in wild animals in southern and central Europe, mainly in foxes. However, there are currently many studies that have described cases of infected domestic dogs in the same areas where previously infected wild animals had been reported [1, 12]. Different diagnostic techniques have been used in these studies, based on the *post-mortem* detection of adult parasites in the pulmonary artery or in the right ventricle [15], or based on serological techniques by the detection of circulating antigens and/or antibodies [1, 22, 23]. For both techniques, the results depicted low prevalences. When the Baermann funnel technique was used, reported prevalences were higher in endemic countries, such as Denmark and Germany [24].

The Angio Detect^TM^ test has a reported sensitivity of 98.1% and a specificity of 99.4% [25, 26]. However, in one canine study it was observed that the earliest positive results using this test were not observed until nine weeks post-infection, and that all studied sera were not positive until another five weeks later [25]. Therefore, the sensitivity to detect early infections seems to be diminished and it is possible that the actual prevalence may have been higher than that reported in this study.

Specific antibodies may be detected by ELISA with a sensitivity and specificity of 85.7% and 98.8%, respectively [27]. They can be detected as early as at three weeks post-infection; however, specific antibodies indicate exposure to the parasite, and seropositive dogs may also be free of parasites (i.e. self-curing or administration of a broad-spectrum macrolactones effective against *A. vasorum*) since they persist up to 63 days after elimination of the parasite [27]. Therefore, this would explain why the percentage seropositivity in other studies where this technique has been used were higher than in those based on the detection of circulating antigens [1, 22, 28].

In this study, the presence of *A. vasorum* in dogs in different regions of Spain has been evaluated, being to date the most extensive study carried out since only reports of the infection exist for the Basque Country to date. The overall prevalence for the regions studied was 1.73%. The highest overall prevalence was recorded in Zone 2 (2.74%) and Zone 1 (1.86%), where the presence of *A. vasorum* has previously been described in wolves, badgers and foxes, sometimes with high prevalences [11–18]. It should be noted that all sampled dogs lived outside or had regular access to outdoor areas, and that the regions with the highest prevalence are areas where vegetation predominates and rainfall is abundant at different times of the year, promoting the presence of intermediate hosts.

In other countries, and using similar diagnostic techniques, the prevalence of canine angiostrongylosis was similar to that recorded in the regions evaluated in the present study (1.99% in Portugal [10] and 1.76% in Hungary [28]). On the other hand, studies in other countries using the same techniques, have reported much lower prevalences (Germany: 0.15% [22]; Romania: 0.19% [1]).

Age is considered a risk factor, with dogs under 18 months-old showing the highest infection risk [29]. In the present study, although the highest prevalence was found in dogs younger than 1-year-old followed by dogs aged 1–4 years-old, the differences were not statistically significant. Furthermore, when evaluated by zones, only significant differences were found in Zone 2 but in other zones no dogs under 1 year (Zones 3 and 5) or dogs under 4 years (Zone 6) were infected. No statistically significant differences were observed by sex, so there seems to be no predisposition between males and females.

## Conclusions

The prevalences recorded in the studied zones indicate that prophylactic measures should be prescribed for dogs that have regular access to the outside and/or contact with wildlife susceptible to the disease. This is especially true in young dogs and puppies in those zones that showed the highest prevalences, which correspond to the wettest and rainiest climates of the country. Taking into account the results obtained, and that *A. vasorum* is considered an emerging parasite in domestic animals, further studies are required to complete the epidemiological map of angiostrongylosis in Spain. In addition, veterinary clinicians should be aware of the importance of this disease in order to carry out appropriate control campaigns and raise awareness among pet owners.

## Data Availability

The datasets supporting the conclusions of this article are included within the article.

## Abbreviations

L1: first-stage larvae
L3: third-stage larvae
L5: fifth-stage larvae
PCR: polymerase chain
ELISA: enzyme-linked immunosorbent assay
